# Could a New COVID-19 Mutant Strain Undermine Vaccination Efforts? A Mathematical Modelling Approach for Estimating the Spread of B.1.1.7 Using Ontario, Canada, as a Case Study

**DOI:** 10.3390/vaccines9060592

**Published:** 2021-06-03

**Authors:** Mattew Betti, Nicola Bragazzi, Jane Heffernan, Jude Kong, Angie Raad

**Affiliations:** 1 Department of Mathematics and Computer Science, Mount Allison University, Sackville, NB E4L 1E2, Canada; 2 Department of Mathematics and Statistics, York University, Toronto, ON M3J 1P3, Canada; bragazzi@yorku.ca (N.B.); jmheffer@yorku.ca (J.H.); jdkong@yorku.ca (J.K.); angieraad15@gmail.com (A.R.); 3 Laboratory for Industrial and Applied Mathematics, York University, Toronto, ON M3J 1P3, Canada; 4 Centre for Disease Modeling, York University, Toronto, ON M3J 1P3, Canada

**Keywords:** mutant strains, emerging infectious diseases, COVID-19 pandemic, mathematical model

## Abstract

Infections represent highly dynamic processes, characterized by evolutionary changes and events that involve both the pathogen and the host. Among infectious agents, viruses, such as Severe Acute Respiratory Syndrome-related Coronavirus type 2 (SARS-CoV-2), the infectious agent responsible for the currently ongoing Coronavirus disease 2019 (COVID-2019) pandemic, have a particularly high mutation rate. Taking into account the mutational landscape of an infectious agent, it is important to shed light on its evolution capability over time. As new, more infectious strains of COVID-19 emerge around the world, it is imperative to estimate when these new strains may overtake the wild-type strain in different populations. Therefore, we developed a general-purpose framework to estimate the time at which a mutant variant is able to take over a wild-type strain during an emerging infectious disease outbreak. In this study, we used COVID-19 as a case-study; however, the model is adaptable to any emerging pathogen. We devised a two-strain mathematical framework to model a wild- and a mutant-type viral population and fit cumulative case data to parameterize the model, using Ontario as a case study. We found that, in the context of under-reporting and the current case levels, a variant strain was unlikely to dominate until March/April 2021. The current non-pharmaceutical interventions in Ontario need to be kept in place longer even with vaccination in order to prevent another outbreak. The spread of a variant strain in Ontario will likely be observed by a widened peak of the daily reported cases. If vaccine efficacy is maintained across strains, then it is still possible to achieve high levels of immunity in the population by the end of 2021. Our findings have important practical implications in terms of public health as policy- and decision-makers are equipped with a mathematical tool that can enable the estimation of the take-over of a mutant strain of an emerging infectious disease.

## 1. Introduction

Infections represent highly dynamic processes [1] that are characterized by evolutionary changes and events that involve both the pathogen and the host [2] and can be understood at two levels, namely the intra- [3] and the inter-host levels [4]. Among infectious agents, viruses, and in particular RNA viruses, have a particularly high mutation rate [5], which is even more relevant in terms of public health control and management [6] considering their short generation times and relatively large population sizes [2]. It is of paramount importance to take into account the mutational landscape of an infectious agent [7], to shed light on its evolutionary capability over time, to be able to capture events leading to a rapid and effective adaptation to the host environment, thus, impacting its fitness and transmissibility to new hosts [8].

Pathogen evolution and recombination can result in escaping the host immune system [9], causing drug failure and leading to the insurgence of anti-microbial drug resistance [10]. Further, this can compromise the effectiveness of existing vaccines making infection prevention and control more challenging [8].

Severe Acute Respiratory Syndrome-related Coronavirus type 2 (SARS-CoV-2) is the infectious agent responsible for the currently ongoing Coronavirus disease 2019 (COVID-2019) pandemic [11]. COVID-19 is a generally mild but sometimes severe and even life-threatening communicable disease [12]. This novel, emerging coronavirus exhibits a constantly and dynamically evolving mutational landscape [13], with a relatively abundant genetic diversity [14] and a high evolution capability over time [15].

As new, potentially more infectious strains of COVID-19 emerge around the world [16], it is imperative to estimate when these new strains may overtake the wild-type strain in different populations. Therefore, we developed a general-purpose framework for estimating the takeover of mutant strains of emerging infectious diseases. In the this study, we used COVID-19 as a case-study; however, the model is also adapted to any emerging pathogen.

## 2. Methods

We extend the model presented in [17]. We maintain a wild-type population and fit cumulative case data to parameterize the wild-type model. The model is quite simple, taking into account the total cases $C_{I}$, known cases $C_{K}$, mild active cases $I_{m}$, and active severe cases $I_{s}$ only. The wild-type model equations first presented in [17] are

(1) $$\begin{array}{cc} \frac{dC_{I}}{d\hat{t}} & =R_{0}M\left(\hat{t}\right)\left(1-\frac{C_{I}}{N}\right)\left(I_{m}+pI_{s}\right)\\ \frac{dC_{K}}{d\hat{t}} & =r\left(1-p_{s}\right)\frac{dC_{I}}{d\hat{t}}+p_{s}\frac{dC_{I}}{d\hat{t}}\\ \frac{dI_{m}}{d\hat{t}} & =\left(1-p_{s}\right)\frac{dC_{I}}{d\hat{t}}-I_{m}\\ \frac{dI_{s}}{d\hat{t}} & =p_{s}\frac{dC_{I}}{d\hat{t}}-I_{s}\; \end{array}$$

where $\hat{t}$ is time in units of infectious lifetime, $R_{0}$ is the basic reproduction number, M(t) is a mitigation function that describes non-pharmaceutical interventions, *N* is the population of the region, *p* is the relative infectiousness of severe cases to mild cases, *r* is the average reporting rate of mild cases, and $p_{s}$ is the probability that a case is severe.

The model comes with a set of assumptions that are discussed in [17], highlighted below:

1. Reporting is relatively consistent.
2. The total population in a region is constant.
3. All severe cases are reported.

We note that the second assumption implies that the model can be applied for short-term projection. Additionally, the third assumption implies that severe cases will always require medical intervention and are, thus, always reported. We also point out that by the first assumption, and by virtue of the model itself, this model assumes that an outbreak is mainly being driven by community transmission.

Using the least squares method, we fit $C_{K}$ to the reported cumulative case data in the province of Ontario and obtained estimates for the total cases and active mild/severe cases. We used data from 12 December 2020 to 11 January 2021 to fit the parameters of the model. The model parameters are listed in Table 1. Validation of this method is found in Appendix A.

With the base parameters, we can then extend the model to account for a more infectious variant. We still fit to the same known infections, $C_{K}$, but we require the wild-type given by $I_{m}$ and $I_{s}$ as well as a mutant strain ${\tilde{I}}_{m}$ and ${\tilde{I}}_{s}$. The full model with both strains is then given by

(2) $$\begin{array}{cc} \frac{dC_{I}}{d\hat{t}} & =R_{0}M\left(\hat{t}\right)\left(1-\frac{C_{I}}{N}\right)\left(I_{m}+pI_{s}+k\left({\tilde{I}}_{m}+p{\tilde{I}}_{s}\right)\right)\\ \frac{dC_{K}}{d\hat{t}} & =r\left(1-p_{s}\right)\frac{dC_{I}}{d\hat{t}}+p_{s}\frac{dC_{I}}{d\hat{t}}\\ \frac{dI_{m}}{d\hat{t}} & =\left(1-p_{s}\right)R_{0}M\left(\hat{t}\right)\left(1-\frac{C_{I}}{N}\right)\left(I_{m}+pI_{s}\right)-I_{m}\\ \frac{dI_{s}}{d\hat{t}} & =p_{s}R_{0}M\left(\hat{t}\right)\left(1-\frac{C_{I}}{N}\right)\left(I_{m}+pI_{s}\right)-I_{s}\\ \frac{d{\tilde{I}}_{m}}{d\hat{t}} & =\left(1-p_{s}\right)kR_{0}M\left(\hat{t}\right)\left(1-\frac{C_{I}}{N}\right)\left({\tilde{I}}_{m}+p{\tilde{I}}_{s}\right)-{\tilde{I}}_{m}\\ \frac{d{\tilde{I}}_{s}}{d\hat{t}} & =p_{s}kR_{0}M\left(\hat{t}\right)\left(1-\frac{C_{I}}{N}\right)\left({\tilde{I}}_{m}+p{\tilde{I}}_{s}\right)-{\tilde{I}}_{s}\;\; \end{array}$$

## 3. Results

### 3.1. Scenario-Based Extrapolation from First Known Cases of B.1.1.7 Variant in Ontario

On 26 December 2020, there were two confirmed cases of the COVID-19 UK variant in Ontario [18]. It is, however, unknown exactly how many cases of the variant existed at at that time, and before or after. Given that the spread of the variant will depend on the number of B.1.1.7 variant infections, we first explore outcomes of our model given different initial conditions of ${\tilde{I}}_{s}$ and ${\tilde{I}}_{m}$.

#### No Vaccination, No Relaxation

**Figure 1:**
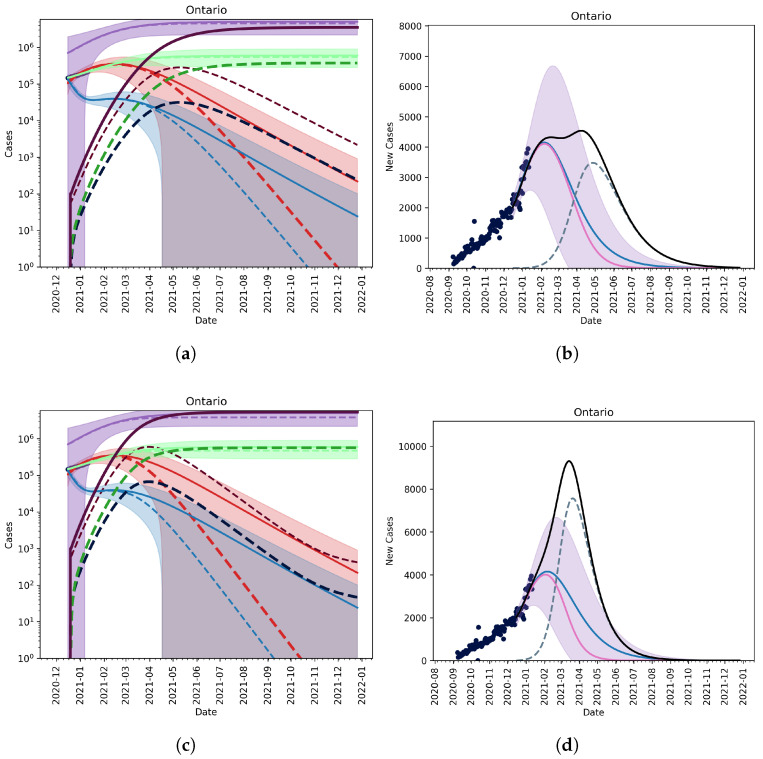
Model fit given different initial conditions for the mutant strain. (**Top row**, (**a**,**b**)) 100 cumulative cases on 26 December 2020. (**Bottom row**, (**c**,**d**)) 1000 cumulative cases on 26 December 2020. (**Left column**, (**a**,**c**)) The active and cumulative cases are shown given the model with (dashed lines) and without (solid lines) the mutant strain (dashed lines). Active mild and severe wildtype infected cases are shown in red and blue. Active and severe mutant infected populations are shown in dark red and dark blue. The cumulative known and total cases of the wildtype and mutant strains are shown in light and dark green, and light and dark purple, respectively. (**Right column**, (**b**,**d**)) The new reported cases per day given the model with (wildtype—solid pink line, mutant—dashed blue line, and total—solid black line) and without (wildtype—solid blue line) the mutant. Ontario reported case data, from September 2020 to January 2021, are also shown (dots).

**Figure 2:**
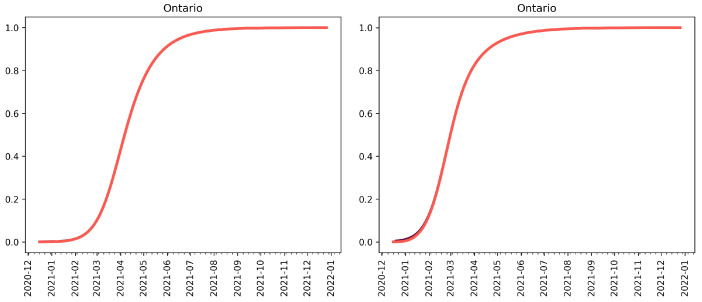
The proportion of active cases of the mutant strain. (**Left**) 60 active cases (100 cumulative cases) on 26 December 2020. (**Right**) 600 active cases (1000 cumulative cases) on 26 December 2020.

Using the fitted average reporting rate, we first assume that there are roughly 60 active cases of the variant in the province at the time, and that the variant is 1.7-times as infectious as the wild-type strain [19]. In Figure 1 (top row), we see that, if the mutant is introduced around Christmas and non-pharmaceutical interventions are continued, the mutant strain cannot saturate until late 2021 and will not become the dominant strain until April 2021 (Figure 2, left panel). This scenario acts as a control as it does not account for vaccination or the relaxation of non-pharmaceutical interventions (NPIs).

In Figure 1 (right panel), we see that, if we assume that there are many cases of the B.1.1.7-strain in Ontario (1000 cumulative cases as of 26 December 2020), by the time we are able to detect two cases, then it is possible for the mutant strain of the SARS-CoV-2 virus to become the dominant strain by March/April 2021 under the current NPI public health measures in place. Figure 2 (right panel) shows that, by early March, the new mutant would account for the majority of active cases in the province of Ontario.

### 3.2. Extension to Vaccination and Relaxation

In Ontario, a vaccination program against COVID-19 has been implemented since 15 December 2020 [20]. Given this, a relaxation of the NPI public health measures may be able to take place. We now study the outcomes of vaccination and relaxation given the existence of the mutant strain in the population. Vaccination and relaxation were implemented in this model in the same way as in [21].

#### 3.2.1. Vaccination without Relaxation

**Figure 3:**
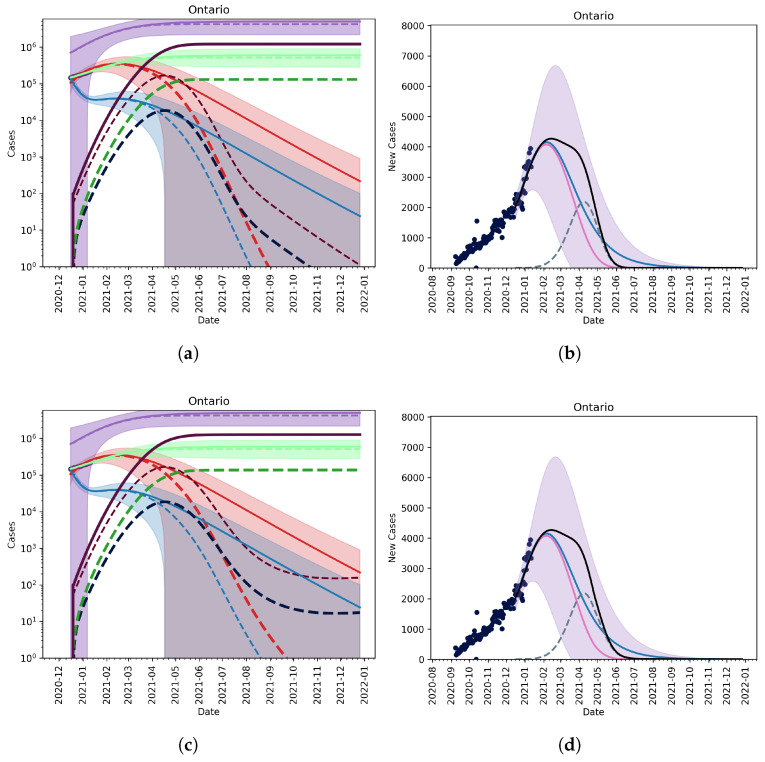

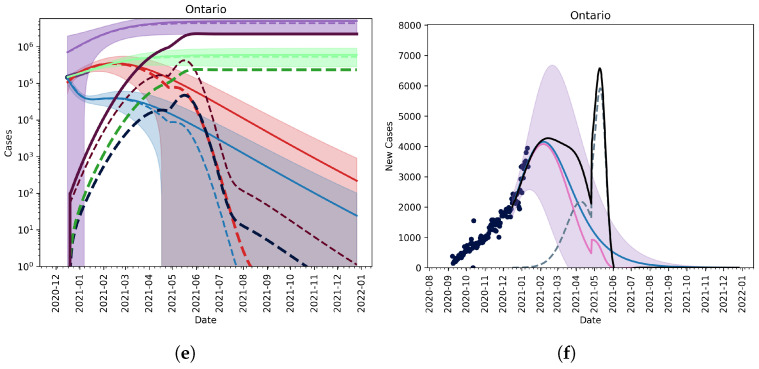
Model fit given vaccination and relaxation. (**Top row**, (**a**,**b**)) vaccination without relaxation, (**middle row**, (**c**,**d**)) vaccination with slow relaxation, and (**bottom row**, (**e**,**f**)) vaccination with fast relaxation. Vaccination assumes that 10% of the population is vaccinated by 31 March 2021 and that 75% of the population is inoculated by the end of 2021. Relaxation allows for rules and behaviours to change in a way that allows for more contact between individuals. We assume that behaviours will eventually lead to pre-February 2020 contact rates between individuals. (Left column) The active and cumulative cases are shown given the model with (dashed lines) and without (solid lines) the mutant strain (dashed lines). Active mild and severe wildtype infected cases are shown in red and blue. Active and severe mutant infected populations are shown in dark red and dark blue. The cumulative known and total cases of the wildtype and mutant strains are shown in light and dark green, and light and dark purple, respectively. (Right column) The new reported cases per day given the model with (wildtype—solid pink line, mutant—dashed blue line, and total—solid black line) and without (wildtype—solid blue line) the mutant. Ontario reported case data, from September 2020 to January 2021, are also shown (dots).

**Figure 4:**
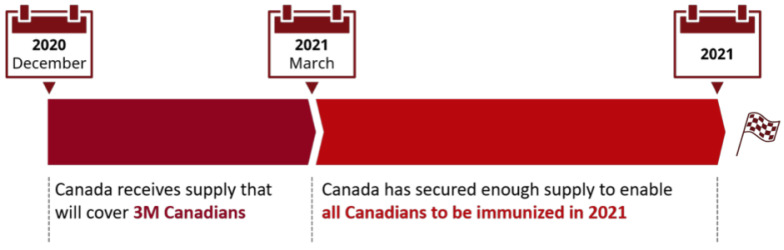
The Federal Government vaccine roll-out plan, adapted from [22].

Figure 3 (top row) shows that a fairly aggressive vaccination plan, namely the vaccination plan outlined in Figure 4 where 10% and 75% of the population are vaccinated by the second quarter, and end of 2021, respectively. We observe that, compared to the results in [21], the new, potentially more infectious strain will require NPIs to be in place much longer. If we maintain and escalate NPIs such that we can reduce transmission by 60%, we can still lower cases to nearly zero by July/August 2021. We can compare this to the results in [21] where we only required a 20% reduction in transmission through NPIs plus vaccination to see a similar effect.

#### 3.2.2. Vaccination and Relaxation

Figure 3 (middle row) shows that the model with a slow, controlled relaxation starting May 2021 resulted in a small outbreak of the B.1.1.7 variant. In Figure 3 (bottom row) we see that, if immediate relaxation to pre-Covid-19 times (i.e., the basic reproduction number of the wildtype strain was increased back to $R_{0}\approx 2.5$) on 1 May 2021, a new outbreak of COVID-19 emerged that was mainly dominated by the new strain.

#### 3.2.3. Fitting an Unknown Mutant

**Figure 5:**
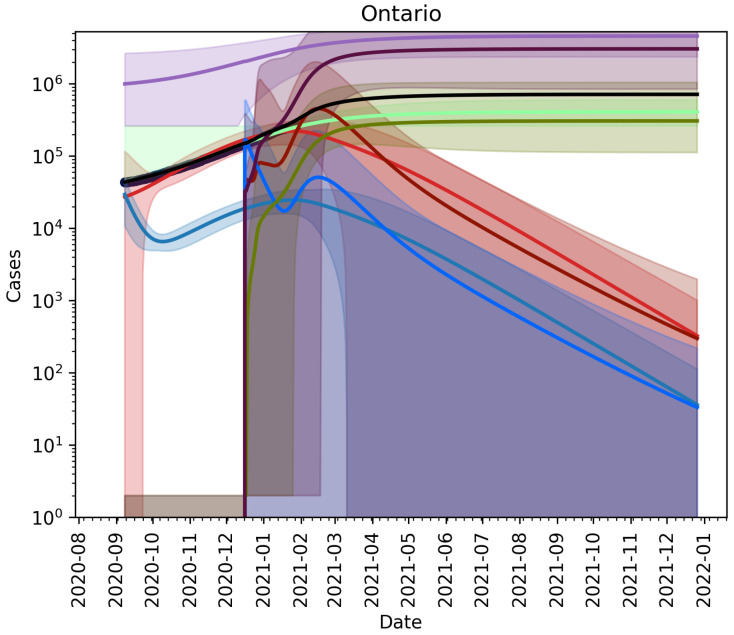
Figure showing the model fit with both the wildtype and mutant. Red is mild cases, blue is severe cases, green is the cumulative reported cases, and purple is the total cases. The light colours are the wildtype, and dark colours are the mutant.

If we try to explain the cumulative reported cases by fitting the model parameters for both the wildtype and mutant, we see that the mutant must have been in Ontario long before it was reported in order to be explained by the data. This predicts a much earlier invasion of the mutant; it becomes dominant by March 2021. Since we only have the total cumulative cases, this fit has a very wide confidence interval. Figure 5 shows that the mutant takes over by the end of February in Ontario. The fit is in-line with what we currently know about the mutant B.117. The fit shows that the mutant is approximately 1.5-times as infectious as the wildtype [23].

#### 3.2.4. Christmas as an Anomaly

**Figure 6:**
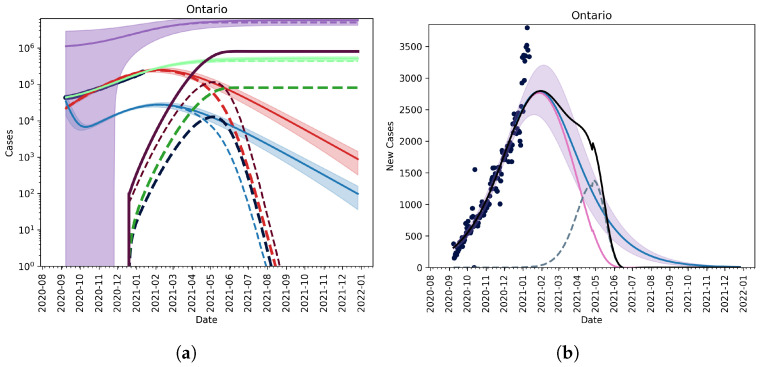
Model fit assuming the period over Christmas to be anomalous, including vaccination and relaxation. Vaccination assumes that 10% of the population is vaccinated by 31 March 2021 and that 75% of the population is inoculated by the end of 2021. Relaxation allows for NPIs to be lifted on 1 May 2021. (**Left column**, (**a**)) The active and cumulative cases are shown given the model with (dashed lines) and without (solid lines) the mutant strain (dashed lines). Active mild and severe wild-type infected cases are shown in red and blue. Active and severe mutant infected populations are shown in dark red and dark blue. The cumulative known and total cases of the wild-type and mutant strains are shown in light and dark green, and light and dark purple, respectively. (**Right column**, (**b**)) The new reported cases per day given the model with (wild-type—solid pink line, mutant—dashed blue line, and total—solid black line) and without (wild-type—solid blue line) the mutant. Ontario reported case data, from September 2020 to December 2020, are also shown (dots).

We note that the reported new cases surrounding the Christmas period may be treated as anomalous data—caseloads fell again in January immediately after the holiday period. Figure 6 plots the model fit given the time period from 9 September 2020 up to 9 December 2020 only, including vaccination and a 1 May relaxation. This approach treats the weeks around Christmas as anomaly. Here, we see that the proliferation of a new, potentially more infectious strain is likely to create a prolonged ‘peak’ in new daily infectious. However, if the vaccine is effective against the new strain, the time to a highly immunized vaccinated population similar to the results reported above, remains largely unchanged.

## 4. Discussion

SARS-CoV-2 is an emerging coronavirus responsible for the still ongoing COVID-19 pandemic. Ontario, Canada, as well as other territories and countries worldwide have experienced multiple waves of COVID-19 and have been struggling to find a difficult compromise between reducing mortality and morbidity in their populations, while also trying to augment economic activity within public health guidelines. One year after the initial outbreak that emerged in December 2019, a number of vaccines against SARS-CoV-2 had been approved, and vaccine distribution had already commenced in Ontario.

New variants of SARS-CoV-2 have emerged worldwide [24,25], and some have been detected in Ontario [18]. The emergence of such variants can reduce the efficacy of current vaccines and vaccination programs, and can affect public health mitigation protocols, i.e., requiring increased and/or sustained restrictions over longer time periods than originally projected. For example, new variants of SARS-CoV-2 may be more transmissible [26,27] or may be less affected by vaccine-induced immunity [24]. Quantification of these affects on vaccination and public health mitigation programs are, thus, needed.

Like other viruses causing widespread transmission in the population, SARS-CoV-2 has mutated many times since its initial insurgence (an outbreak of pneumonia of unknown etiology that occurred in Wuhan, province of Hubei, mainland China). Based on its genomic profile, SARS-CoV-2 can be subdivided into various genetic groups, known as clades. A set of specific mutations would enable researchers to distinguish between the viral groups currently dominating and circulating worldwide. These groups are generally called lineages, even though the precise nomenclature and the taxonomic hierarchy of SARS-CoV-2 are still under debate and, generally speaking, classifying viral variety and diversity is a challenging task [28,29].

Mutations arise spontaneously as a consequence of a complex, multi-factorial series of macro- and micro-evolution processes as well as the result of selection pressures [16]. However, some of these mutations (termed as “variants of concern”, VOCs) may be particularly clinically meaningful, especially from the public health perspective, as they may be associated with higher forces of infection, transmissibility, and mortality [16].

In particular, since December 2020, some VOCs have been reported by national public health authorities to the World Health Organization (WHO), including VOC-202012/01 (also known as lineage B.1.1.7, commonly referred to as the “B.1.1.7 variant” or the “British variant”), 20I/501Y.V2 (known as lineage B.1.351, commonly termed as the “South African variant”), and lineage B.1.1.28 (known as the “Brazilian variant”). Other variants are under investigation and strict follow-up from international public health bodies, including the “Japanese variant” (variant P.1, lineage B.1.1.28) and the “USA variant” (L452R). This topic is constantly under flux as identifying the impact of a variant is of paramount importance. Once introduced in the population, a highly transmissible variant could become increasingly prevalent, thus, leading to the replacement of the original wild strain and making infection control and management particularly difficult.

We have extended our previous modelling framework to a study of SARS-CoV-2 variants and their effect on vaccination and public health mitigation program needs. Our findings have important practical implications in terms of public health as policy- and decision-makers are equipped with a mathematical tool enabling the estimation of the take-over of a mutation strain of an emerging infectious disease, such as the previously mentioned VOCs. Our results demonstrated that, in the context of under-reporting and the current case levels, a variant strain was unlikely to dominate until March/April 2021. However, the current NPIs in Ontario will need to be kept in place for longer even with vaccination in order to prevent another outbreak. Our fitted model in Figure 5 showed that reopening in Ontario could have detrimental effects due to the possibility that the more infectious mutant is more widespread than thought.

Additionally, we find that the proliferation of a variant strain in Ontario will mostly likely be observed by a lengthened flat peak of reported daily cases (see Figure 1 (top row), and Figure 3). If vaccine efficacy is maintained across strains, then it is still possible to acquire a high enough level of immunity to protect the entire population by the end of 2021 (see Figure 3). A limitation of this model is that it does not account for the importation of cases, which could prolong outbreaks; however, with the new rules set in place by the Government of Canada surrounding international travel [30], the practical effects of importation are low.

## Figures and Tables

**Table 1:** Table of the model parameters from the fit; used as a base for extensions. We extend the model with the parameters using hypothetical scenarios for vaccination, relaxation, and the emergence of a variant strain. These give the mean values of the model parameters with one standard deviation.

Parameter	Fitted Value
$R_{0}$	2.41±0.59
*p*	0.25±0.21
*r*	0.006±0.02
*k*	0.40±0.22
*d*	0.21±0.29

**Figure A1:**
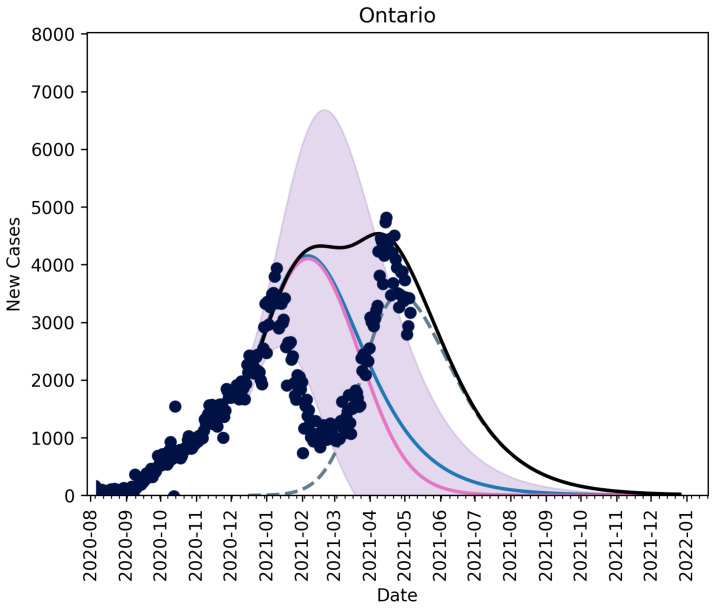
Figure validating model fitting and scenario building. The model is fit to data from 12 December 2020 to 11 January 2021. We augment the data with data from 12 January 2021 until 7 May 2021. We see that the hybrid data and scenario approach properly detected peaks out to four months in advance. Between January and April 2021, Ontario went through multiple phases of lockdown and relaxation of non-pharmaceutical interventions, which the model does not capture (as they were unknowable at the time). The peak detection makes the model a valuable tool for long-term planning. Thee pink line is the continuation of the fit, the blue line is the new wildtype trajectory when the mutant scenario is introduced, and the dashed line is the mutant strain. The black line is the combination of wildtype and mutant. The black line is the total new cases per day, and this is what should be compared to the data as the new cases per day is reported as a total. The shaded region is the 95% confidence interval from the fit.

**Figure A2:**
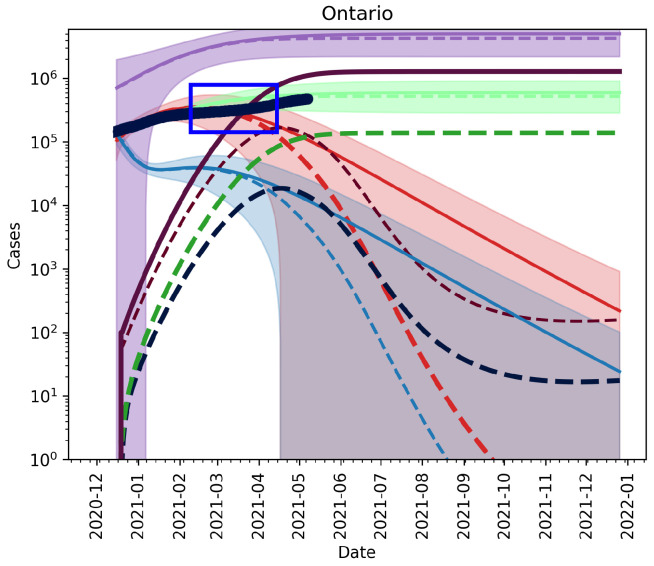
Figure 6a augmented with data up to 7 May 2021. The model is still only fit to data up to 11 January 2021. We see that the line fit (cumulative known wildtype cases) is still relatively close 4 months out. As before, green represents the cumulative known cases, purple is the total incidence, red is active mild cases, and blue is active severe cases. Dark lines are mutant compartments, and light colours are wildtype. Dashed lines are the extended scenario, and solid lines the original fit without a separation of wildtype and mutant. The light green line is what is being fit by the model. We see some anomalous behaviours in the extension due to the period of lockdown–relaxation cycles in Ontario. While the log scale obfuscates some detail, the consequence of these lockdown–relaxation cycles not being present in thee model causes an over-estimation on the order of $10^{4}$ cases.

**Figure A3:**
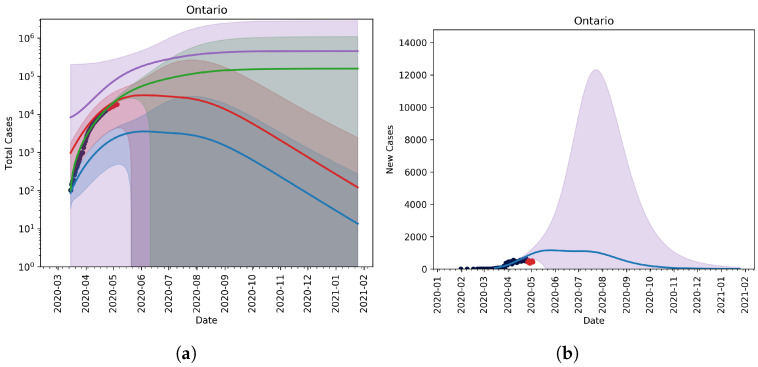
The model projections from the first wave of COVID-19 in Ontario. The red dots are data not used in the model fitting. The green line is the cumulative known cases, purple is the total incidence, red is active mild cases, and blue is active severe cases. In the right panel (**b**), we see the new cases per day. We see that the model performs well for at least two weeks when non-pharmaceutical interventions remain relatively constant. The shaded region is the 95% confidence interval from the fit. The 95% confidence interval was able to detect the presence of a second wave in the fall.

## Data Availability

Data from: https://www.canada.ca/en/public-health/services/diseases/2019-novel-coronavirus-infection.html; Model implemetation available at: https://github.com/mbetti-phd/Covid-SimpleModel.

## Appendix A

In this appendix, we provide some supplementary results that show validation of the model with further data. We note that, in Ontario between January and April 2021, there was a lockdown, followed by a relaxation, followed by another lockdown. This anomalous behaviour was not modeled, making this time period a poor choice for model validation.

Figure A1 reproduces Figure 1b with data points added from 1 January 2021 to 7 May 2021. We see that the model does not capture the period of lockdown and relaxation present in Ontario. This is expected as we did not account for this in our model. To the credit of the model, we still correctly detect the peak 4 months in advance to within a few hundred cases.

Figure A2 shows that the model maintains a decent forecast of cumulative cases when the data is extended to include points up to 15 April 2021. The discrepancy, highlighted by the blue box, shows a cycle of lockdown–reopening–lockdown that was enforced in Ontario but was not present in the model.

We also highlight the strength of the model by reproducing two figures from [17] here. In Figure A3, we see a fit of the model from the first wave of COVID-19 in Ontario. The red data points are data that are *not* used in fitting. We see that the model projections are reliable for at least two weeks when non-pharmaceutical interventions remain relatively constant.
